# Targeting CXCR1 on breast cancer stem cells: signaling pathways and clinical application modelling

**DOI:** 10.18632/oncotarget.6234

**Published:** 2015-10-26

**Authors:** Laura Brandolini, Loredana Cristiano, Alessia Fidoamore, Maria De Pizzol, Erica Di Giacomo, Tiziana Marilena Florio, Giuseppina Confalone, Angelo Galante, Benedetta Cinque, Elisabetta Benedetti, Pier Adelchi Ruffini, Maria Grazia Cifone, Antonio Giordano, Marcello Alecci, Marcello Allegretti, Annamaria Cimini

**Affiliations:** ^1^ Dompé Farmaceutici SpA, Via Campo di Pile, L'Aquila, Italy; ^2^ Department of Life, Health and Environmental Sciences, University of L'Aquila, L'Aquila, Italy; ^3^ Dompé Farmaceutici SpA, Via Santa Lucia, Milano, Italy; ^4^ National Institute for Nuclear Physics (INFN), Gran Sasso National Laboratory (LNGS), Assergi, Italy; ^5^ Department of Medicine, Surgery and Neuroscience, University of Siena, Siena, Italy; ^6^ Sbarro Institute for Cancer Research and Molecular Medicine and Center for Biotechnology, Temple University, Philadelphia, PA, USA

**Keywords:** preclinical MRI

## Abstract

In breast cancer it has been proposed that the presence of cancer stem cells may drive tumor initiation, progression and recurrences. IL-8, up-regulated in breast cancer, and associated with poor prognosis, increases CSC self-renewal in cell line models. It signals via two cell surface receptors, CXCR1 and CXCR2. Recently, the IL-8/CXCR1 axis was proposed as an attractive pathway for the design of specific therapies against breast cancer stem cells. Reparixin, a powerful CXCR1 inhibitor, was effective in reducing *in vivo* the tumour-initiating population in several NOD/SCID mice breast cancer models, showing that the selective targeting of CXCR1 and the combination of reparixin and docetaxel resulted in a concomitant reduction of the bulk tumour mass and CSC population. The available data indicate that IL-8, expressed by tumour cells and induced by chemotherapeutic treatment, is a key regulator of the survival and self-renewal of the population of CXCR1-expressing CSC. Consequently, this investigation on the mechanism of action of the reparixin/paclitaxel combination, was based on the observation that reparixin treatment contained the formation of metastases in several experimental models. However, specific data on the formation of breast cancer brain metastases, which carry remarkable morbidity and mortality to a substantial proportion of advanced breast cancer patients, have not been generated. The obtained data indicate a beneficial use of the drug combination reparixin and paclitaxel to counteract brain tumour metastasis due to CSC, probably due to the combined effects of the two drugs, the pro-apoptotic action of paclitaxel and the cytostatic and anti-migratory effects of reparixin.

## INTRODUCTION

Breast cancer is the leading cause of death in women between the age of 35 and 55. Despite the significant improvements in diagnosis and treatments, breast cancer recurrences and metastasis remain the most common cause of mortality following resection [1]. About 10–30% of patients with disseminated breast carcinoma develop metastasis in the CNS, generally with a median survival of 1 year [2–3], despite the standard treatments. Patients with HER2 + or triple negative breast cancer (TNBC) are more likely to develop brain metastasis [4–6].

In breast cancer, like in other solid tumour types, the presence of a small subpopulation of highly tumorigenic cells within the bulk of tumour cells has suggested that a small set of cancer stem cells (CSC) may drive tumor initiation, progression and recurrences. Several preclinical and clinical studies [7–8] contributed to demonstrate that the CSC population is relatively resistant to radiation therapy and standard chemotherapeutics, thus pointing to the need for new targeted treatments.

Interleukin (IL)-8, known to be up-regulated in several cancers, including breast cancer, and associated with poor prognosis, increases CSC self-renewal in cell line models *in vitro* [9–10]. IL-8 signals via two cell surface G-protein– coupled receptors, CXCR1 and CXCR2. The IL-8/CXCR1 axis was recently proposed as an attractive pathway for the design of specific therapies against breast cancer stem cells. In fact, CXCR1 was found, to be overexpressed in a highly tumorigenic subset of cells expressing the breast stem cell marker ALDH1 in a series of breast cancer cell lines [11] as well as on mammospheres grown *in vitro* from patients' tumor samples [9]. Moreover, the blockade of the receptor resulted in a significant decrease of the overall CSC population both *in vitro* and *in vivo*. Other studies confirmed a role of IL-8 in the regulation of CSC activity in patient-derived breast cancer cells isolated from metastatic ascites, pleural effusions, and primary invasive cancers, thus pointing toward a clear correlation between IL-8 concentration in metastatic fluids and mammosphere formation [9, 12].

Further studies showed that the effects of CXCR1 on CSC are mediated by the FAK/AKT pathway and that cells with constitutive activation of the FAK/AKT (associated with PTEN deletion or FAK overexpression) are invariably resistant to anti-CXCR1 therapy [11].

In the same work reparixin (formerly repertaxin), a powerful small molecular weight CXCR1 inhibitor, was tested in several NOD/SCID mice breast cancer models showing that selective targeting of CXCR1 may effectively reduce *in vivo* the tumour-initiating population and that the combination of reparixin and docetaxel, one of the most effective chemotherapeutic currently available for the treatment of breast cancer patients, resulted in a concomitant reduction of the bulk tumour mass and CSC population. Similarly to the known chemoresistance of the CSC population, docetaxel, when administered alone, did not affect the CSC population, resulting in a relatively small CSC increase in some cases. These observations were reproducible across the two largely non-overlapping breast CSC (BCSC) populations, i.e., ALDH^+^ and CD44^+^/CD24^−^, that can be found in breast cancer [11]. The current bulk of available data clearly outlines that IL-8, expressed by tumour cells and induced by chemotherapeutic treatment, is a key regulator of the survival and self-renewal of the small population of CXCR1-expressing CSC, thus setting the premises for important clinical studies. Consequently, the present investigation on the mechanism of action of the combined treatment with reparixin and paclitaxel (another fundamental drug in the treatment of breast cancer) was based on the observation that, in previous experiments, the effects of the drug combination on the bulk population reduction was significantly higher than the effects of docetaxel alone. This fact can not simply be explained by the action on the chemotherapy-resistant CSC due to the paucity of the CXCR1-expressing cells within the bulk [11]. In fact, reparixin treatment clearly contained the formation of metastasis in several experimental models [11], but specific data on the formation of breast cancer brain metastasis, which carry remarkable morbidity and mortality to a substantial proportion of advanced breast cancer patients, have not been generated.

In this work we have studied the effects of reparixin, alone or in combination with paclitaxel, on mammospheres derived from a highly aggressive triple-negative breast cancer cell line MDA-MB231 and also in a murine model of breast cancer metastasis development into the brain using the same cell line. The murine model was implemented through two experimental settings: the first one was implemented on an early metastatic growth model, while the second one was implemented on an established brain metastases one. This study was conducted following tumour appearance, growth and localization in vehicle and treated animals by means of *ex vivo* high-resolution MRI, histochemical and immunohistochemical examinations. The obtained data, confirming the effects of reparixin on the CSC population, point toward a beneficial use of the drug combination reparixin and paclitaxel to counteract brain tumour metastasis. This is probably due to the combined effects of the two drugs, the pro-apoptotic action of paclitaxel and the cytostatic and anti-migratory effects of reparixin.

## RESULTS

### Mammospheres formation and characterization from MDA-MB231

**Figure 1:**
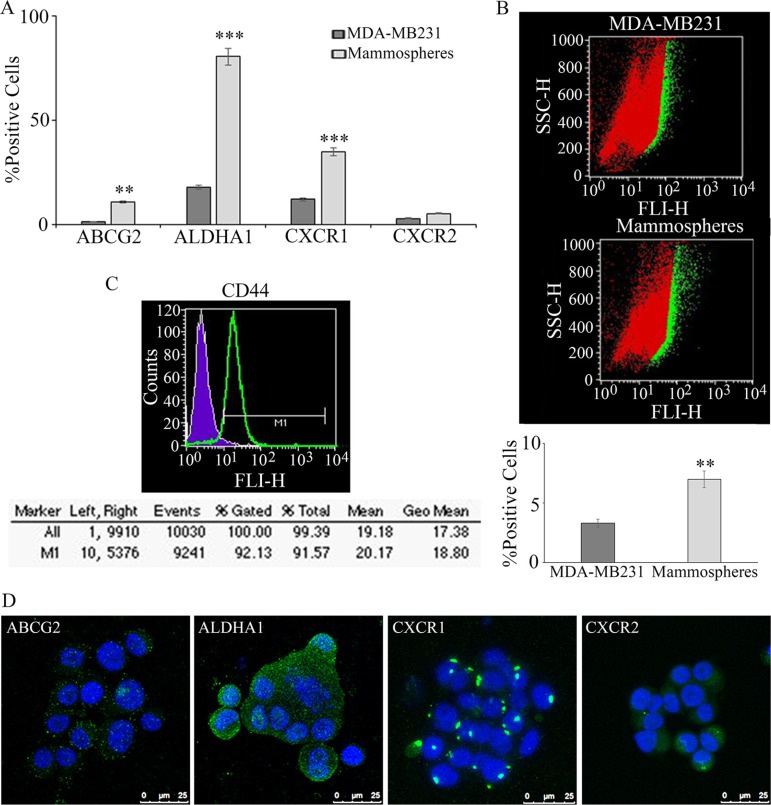
Mammospheres formation and characterization from MDA-MB231: breast tumor stem cell marker enrichment, with respect to the starting cell line, such as ABCG2 and ALDHA1, CXCR1 and CXCR2, evalutated by cytofluorimetry, is reported in (A) In **(B)** the increase of the stem cell population in the mammospheres is confirmed by the Aldefluor assay, measuring ALDHA1 activity. On purified mammospheres CD44 was also measured by cytofluorimetry **(C)** Data are mean ± SE of three different experiments. **p ≤ 0.005; ***p ≤ 0.0005. In **(D)** immunofluorescence for ABCG2 and ALDHA1, CXCR1 and CXCR2 in purified mammospheres. Bar = 25 μm.

In breast cancer, the expression of stem cell markers such as CD44, ATP-binding cassette sub-family G member 2 (ABCG2) and aldehyde dehydrogenase A1 (ALDHA1) can be used to selectively isolate a cell population enriched in CSC. In Figure 1A, ABCG2 and ALDHA1 enrichment, with respect to the starting cell line, is reported. As shown in the panel, the mammospheres isolation and purification procedure is paralleled by a progressive significant increase of the ALDHA1 and ABCG2 stem cell marker expression (about 80% and 11%, respectively). The relative increase of the CSC population in the mammospheres is also confirmed by the Aldefluor assay, measuring ALDH enzymatic activity (Figure 1B). Different percentages of enrichment are apparent considering ALDHA1 as antigen or as enzymatic activity (about 4 folds and 2 folds, respectively) suggesting that not only expression of the ALDHA1 isoform but also overall enzymatic activity of ALDH is increased in the mammospheres. Though the fraction of CXCR1 expressing cells detected in MDA-MB231 was relatively high (> 10%, Figure 1A) as compared to data reported in other breast cancer cell lines, in agreement with previous results [11], a significant enrichment of the CXCR1 positive cells was observed. The fraction of CXCR2 positive cells, present at a low extent in the bulk population, was found to increase in the purified mammospheres, even if not significantly. On purified mammospheres by clonal selection, CD44, the most consistently used biomarker, together with Cd24^low^, to identify and characterize the breast cancer stem cell (BCSC) phenotype [13], was also measured by cytofluorimetry, indicating that about 90% of the purified stem cells were CD44^+^ (Figure 1C). In these series of experiments, since it is known that MDA-MB231 cells are for the 80% CD44^+^/Cd24^low^ [14], on purified mammospheres only CD44 was assayed. The cytofluorimetric data were further confirmed by the immunolocalization experiments. In Figure 1D, the localization of stemness markers in purified tumorspheres is shown. It is possible to observe that almost all cells in a field express ABCG2, ALDHA1, CXCR1, while only few cells express CXCR2.

### Effect of reparixin treatment on MDA-MB231 and on tumorspheres viability and apoptosis

**Figure 2:**
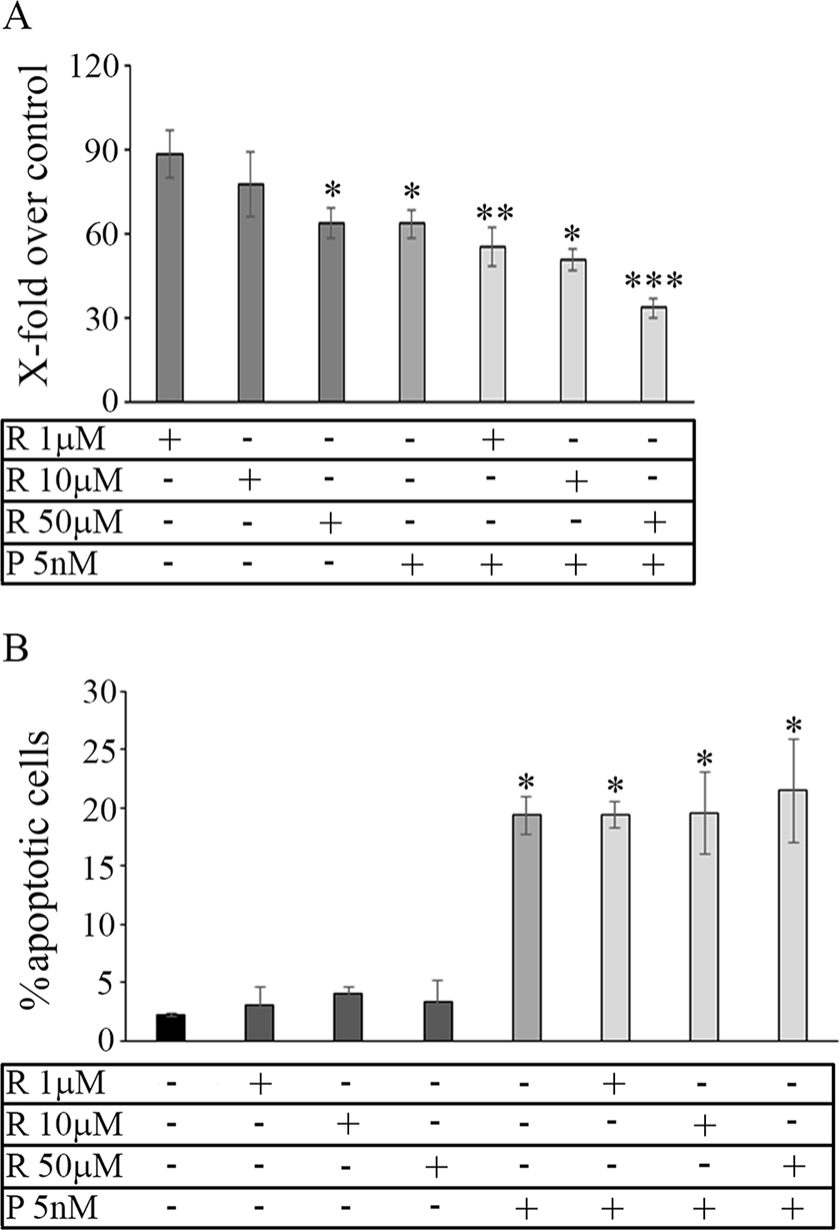
Cell viability: mammosphere viability (A) in control and treated conditions Data are expressed as percentage of the respective control. In **(B)** cytofluorimetric apoptosis detection in purified mammospheres in control and treated conditions. Data are mean ± SE of three different experiments. *p ≤ 0.05; **p ≤ 0.005; ***p ≤ 0.0005. R: reparixin; P: paclitaxel; R + P: reparixin + paclitaxel.

The dose-effect of reparixin on MDA-MB231 was evaluated treating cultured adherent cells at different drug concentrations (1 μM-3 mM) for 72 h. A moderate effect on viability was observed only after 3 days of treatment at high drug concentrations (1–3 mM, not shown). A more pronounced effect on cell viability was observed when MDA-MB231-derived tumorspheres were treated with reparixin and/or paclitaxel. In particular, as shown in Figure 2A, reparixin (R) decreased cell viability in a dose-dependent manner up to about 60% of the control, reaching a significant efficacy at 50 μM. At the concentration of 5 nM paclitaxel (P) induced a comparable decrease of the cell viability, while the combination of the two drugs (R + P) at the above concentrations showed an additive effect.

In contrast with previous observation in other breast cancer cell lines reparixin did not significantly induce apoptosis (Figure 2B) in the range of 1–50 μM, whereas about 20% of apoptotic cells were counted after an equivalent time of paclitaxel 5 nM treatment. No influence of the combination of 5 nM paclitaxel treatment with 50 μM reparixin on the induction of apoptosis in the mammospheres by MDA-MB231 was observed.

In order to investigate potential synergistic effect of the reparixin/paclitaxel combination, a lower (10 mμM) concentration of reparixin was used in the subsequent experiments.

### Tumorspheres number and size

**Figure 3:**
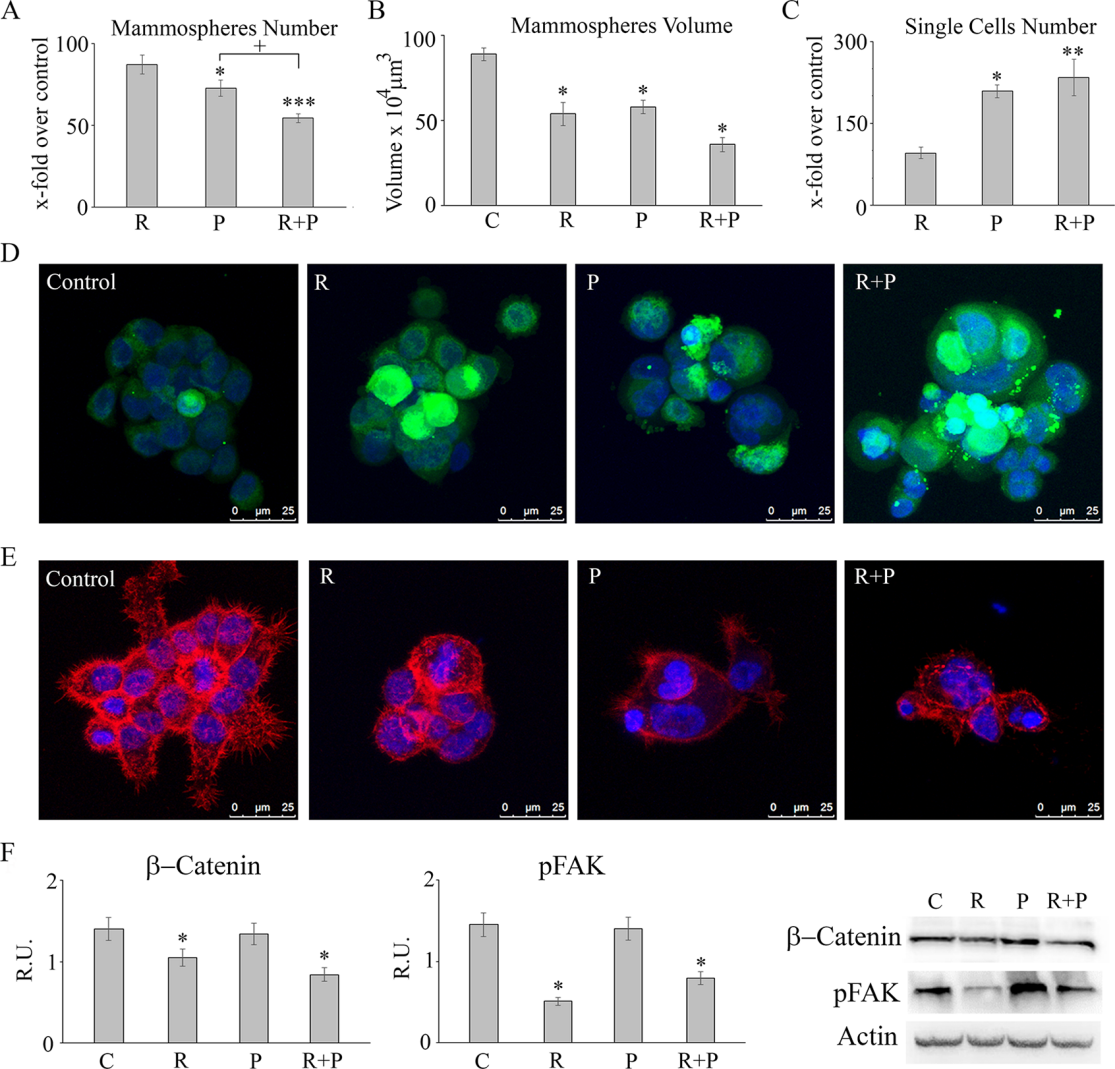
Tumorsphere, number, size, proliferation and microfilament/fokal adhesion organization: In (A-C) for mammospheres number, and for single cells, data are expressed as X-fold of the respective control, while volume is expresed in μm3 Data are mean ± SE of three different experiments. *p ≤ 0.05; **p ≤ 0.005; ***p ≤ 0.0005; +, *p*≤ 0.05. In **(D)** CFSE staining in control and treated mammospheres. Bar = 25 μm. In **(E)** falloidin-TRITC decoration of microfilaments in control and treated mammospheres. Bar = 25 μm. In **(F)** a representative western blotting and relative densitometric analysis, expressed as relative units, for p-FAK and β-catenin in control and treated mammospheres. Data are mean ± SE of three different experiments. *p ≤ 0.05. C: control: R: reparixin 10 μM; P: paclitaxel 5 nM; R + P: reparixin 10 μM + paclitaxel 5 nM.

Tumorspheres were also investigated for number and size upon treatments (Figure 3A and 3B). Reparixin, differently from paclitaxel, did not show a significant ability to reduce tumorsphere number, but the combined treatment showed a synergistic effect (Figure 3A). Both reparixin and paclitaxel treatment reduced the volume of the tumorspheres, with the combined treatment showing a significant additive effect (Figure 3B). Among the two treatments, only paclitaxel showed the ability to increase the number of single cells (dead cells, as determined by counting cells by Trypan Blue exclusion) detached from the spheres, thus supporting a different mechanism of action of the two drugs (Figure 3C).

### Tumorspheres proliferation and migration

Figure 3D reports also the CFSE staining of control and treated tumorspheres. CFSE is a vital staining that allows to trace not proliferating cells, which retain the staining, compared to proliferating ones, in which the staining is diluted. While paclitaxel increases the number of dead cells, in the presence of reparixin a clear increase of the number of not proliferating cells was observed. Both phenomena occur when tumorspheres were exposed to the drug combination. Tumor stem cells were analyzed for microfilaments of lamellipodia/philipodia organization by phalloidin TRITC staining (Figure 3E). As shown, reparixin was able to completely abrogate philopodia/lamellipodia formation in tumorspheres both as single agent or in combination with paclitaxel, being this effect much less evident in paclitaxel treated cells. Coherently with this effect on microfilaments and with the results previously reported in other tumor cell lines [11], the western blotting analysis showed a marked decrease of the active form of the protein of focal adhesions (p-FAK), strongly involved in the migration capability of cells, in the presence of reparixin, thus confirming that also in MDA MB231-derived mammospheres, reparixin action is mediated by inhibition of the FAK/AKT pathway that is not affected by paclitaxel. The combined treatment showed the same effect of reparixin alone thus confirming that the mechanism of action of the two drugs can concur to an effective control of tumor initiating cancer cells growth and invasiveness. It has been previously reported that the *in vitro* and *in vivo* inhibition of FAK decreased self-renewal capacity, by decreasing the levels of Wnt3a and β-Catenin, thus demonstrating a novel FAK-Wnt axis regulating breast cancer stem cell activity and indicating the FAK-Wnt axis as promising target to eradicate self-renewal capacity and progression of human breast cancers [15]. In agreement, in our experimental conditions, the proliferation pathway Wnt-Δ-Catenin, appeared decreased only in the presence of reparixin, thus suggesting a cytostatic effect of reparixin on cancer stem cells by modulating the FAK-Wnt axis (Figure 3F).

### Effect of reparixin and paclitaxel on tumorspheres cell cycle

**Figure 4:**
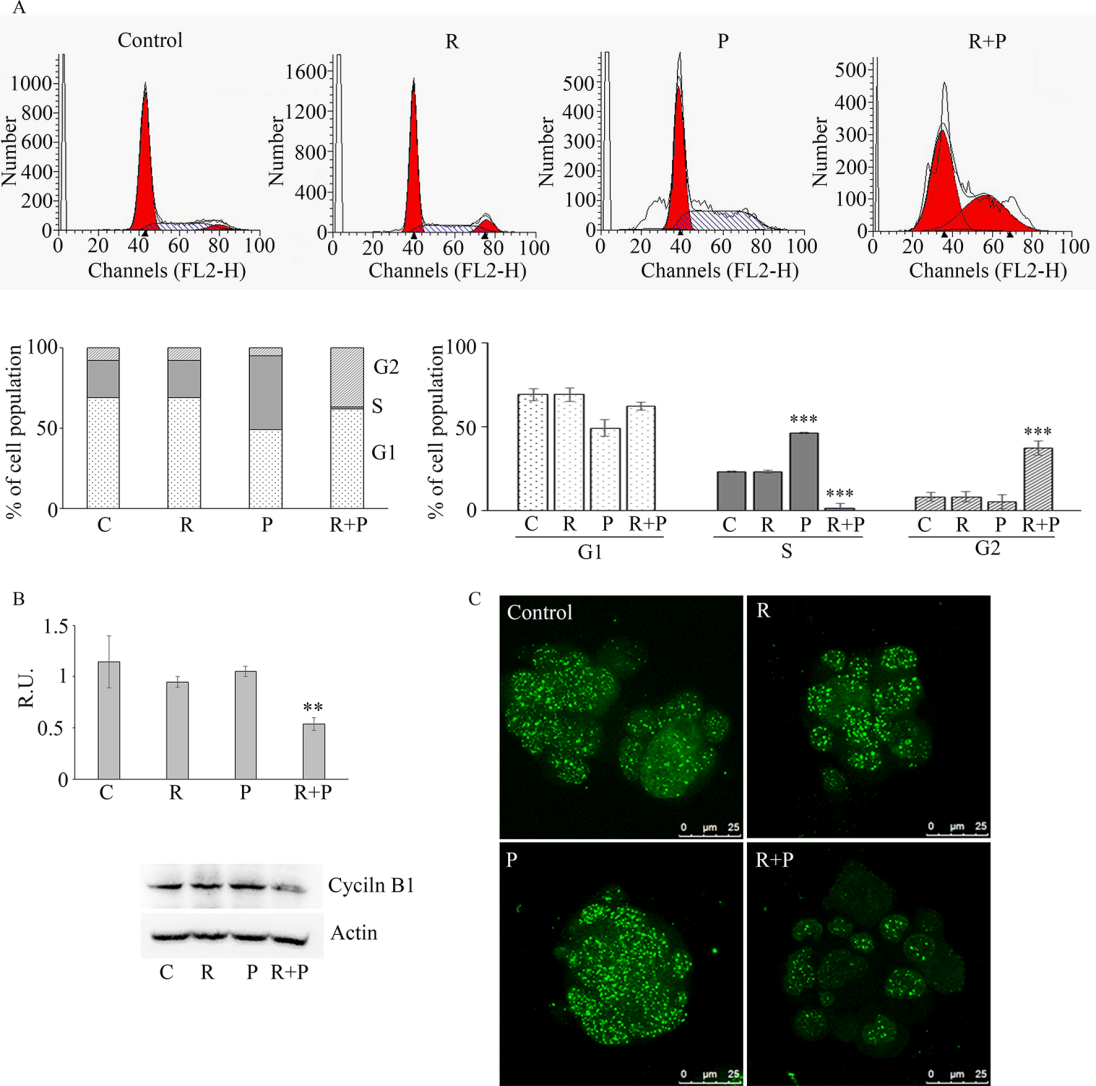
In (A) cytofluorimetric analyisis of cell cycle on control and treated mammospheres Data are mean ± SE of three different experiments. ***p ≤ 0.0005. In **(B)** a representative western blotting and relative densitometric analysis, expressed as relative units, for cyclin B1 in control and treated mammospheres. Data are mean ± SE of three different experiments. **p ≤ 0.005. In **(C)** immunofluorescence analysis for cyclin B1 in control and treated mammospheres, Bar = 25 μM. C: control: R: reparixin 10 μM; P: paclitaxel 5 nM; R + P: reparixin 10 μM + paclitaxel 5 nM.

The effect of reparixin and paclitaxel treatment on the cell cycle was evaluated by cytofluorimetry (Figure 4A). The figure shows a shift of the tumor cells in S phase upon paclitaxel treatment and a block of cells in G2 phase upon combined treatment. Even if paclitaxel is reported to have a cytostatic effect, we can speculate that, being sphere cultures enriched in CSC, up-regulation of DNA synthesis upon paclitaxel treatment account for cells with CSC features that are resistant to therapy by definition. In addition, this may also be due to the release of IL-8 from apoptotic cells, which in turn stimulates cell proliferation. In fact, it is known that several factors are synthesized and secreted during the apoptotic process when the bulk tumor cells are targeted by chemotherapy. Interestingly, it has been reported that chemotherapy may also induce IL-8 production in injured cells [11]. This may partially account for the increase of CSCs proliferation after chemotherapy. The block in G2 phase after the combination therapy confirms reparixin activity on CSC, being reparixin able to eliminate cell with CSC features. The combined R + P treatment acted in a synergistic manner, being able to abolish the IL-8 effects (by blocking CXCR1) and by inducing a block of the cells in the G2 phase, while the S phase almost completely disappeared (Figure 4A). This suggests that the addition of reparixin to cytotoxic chemotherapy may block this proliferative effect by targeting the CSC population.

Figure 4B shows the western blotting analysis for cyclin B1, responsible for the cell cycle progression G2/M. In agreement with the cytofluorimetric data, cyclin D1 was not affected by the treatments (data not shown), while the inhibition of cyclin B1 expression by the combined treatment was coherent with the observed arrest in the G2 phase (Figure 4B). In agreement, in our experimental model high cyclin B1 nuclear staining (evalutated by the merge with DAPI nuclear staining, not shown) is observed in mammospheres (Figure 4C), staining that, after the combined treatment, appeared strongly decreased. High levels of nuclear cyclin B1 are associated with high tumor grade, larger tumor size and higher metastasis probability, therefore a high level of cyclin B1 is considered a predictor of poor prognosis. The latter results show that the blockage of CXCR1-signalling in tumorspheres not only directly reduces the proliferation and invasiveness of cancer stem cells but that also potentiates the anti-tumor effect of the standard chemotherapy with paclitaxel by a synergistic action leading to downregulation of cyclin B1.

### Effect of reparixin and paclitaxel on tumorspheres cell senescence

**Figure 5:**
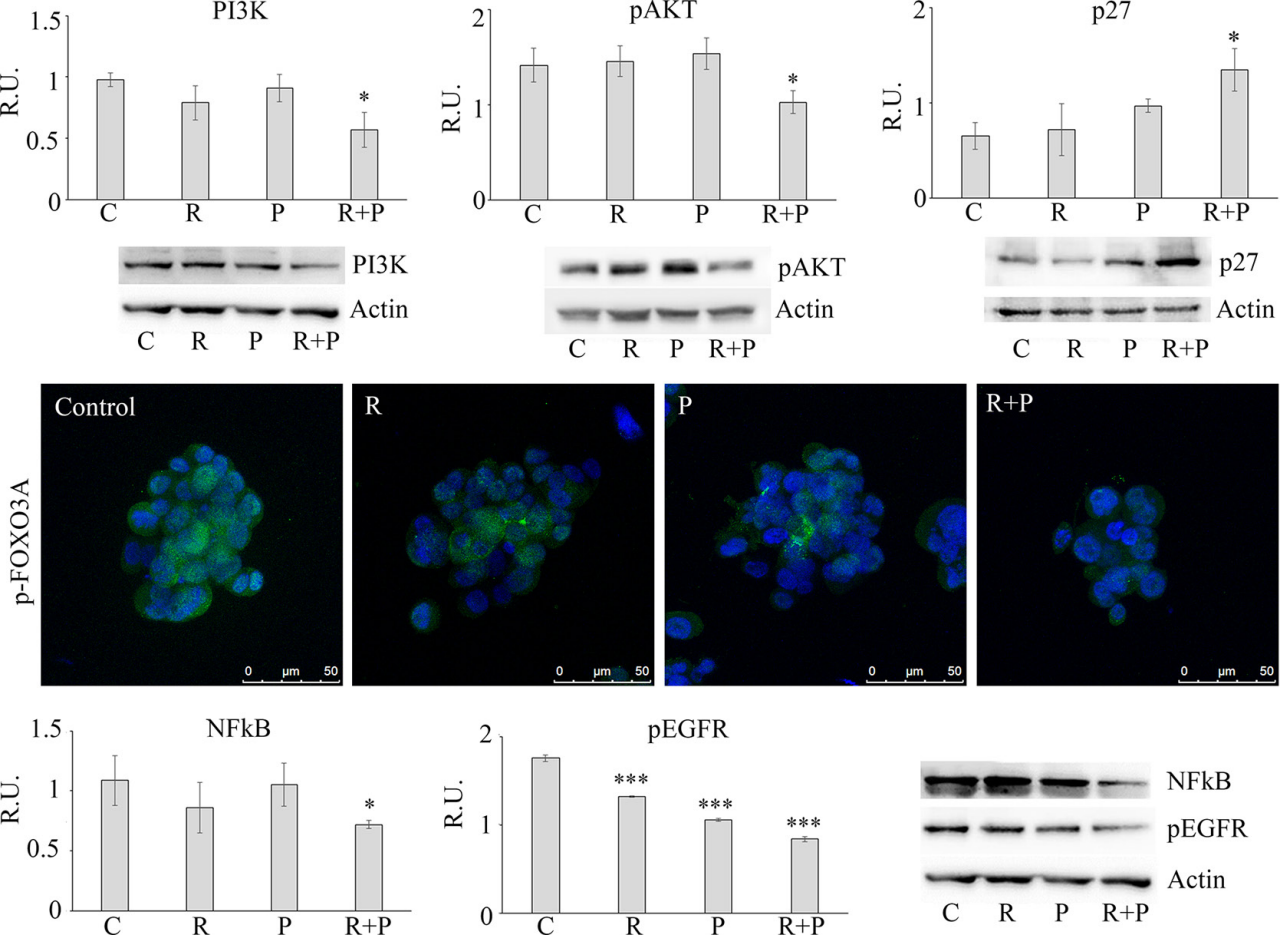
Representative western blotting and relative densitometric analysis, expressed as relative units, for PI3K, p-AKT, p27, NFKB, p-EGFR in control and treated mammospheres Data are mean ± SE of three different experiments. *p ≤ 0.05; ***p ≤ 0.0005. p-FOXO3A was analyzed by immunolocalization of the inactive phosphorylated form. Bar = 50 μm. C: control: R: reparixin 10 μM; P: paclitaxel 5 nM; R + P: reparixin 10 μM + paclitaxel 5 nM.

Since it has been recently described an involvement of the PI3K-Akt-FOXO3-p27 pathway [16] in cellular senescence, in order to better understand the differences in cell cycle phases induced by the different treatments, this pathway was further investigated (Figure 5). In agreement with the synergistic effect of R + P in inducing a G2/M arrest of cell cycle, it appeared that the combined treatment was able to induce cell senescence by decreasing PI3K-Akt activation paralleled by a decrease of the cytosolic p-FOXO3A (inactive) and by an increase of p27, thus suggesting that cells blocked in G2/M by the two drugs may undergo to senescence. To further explore the cell senescence pathway, we studied the nuclear factor NFkB that has recently been proven to have a role in antagonizing senescence [17], in addition to being one of the effector in IL-8 signaling. Reparixin alone showed a trend in reducing NFkB levels, while paclitaxel was uneffective. The combination of the two drugs showed a significant effect in reducing NFkB levels (Figure 5). Finally, since the interactions between the IL-8/CXCR1/2 and HER signaling in the regulation of BCSCs has been demonstrated [9], EGFR receptor phosphorylation was investigated upon the different treatments. In agreement with the previous observations, our data show that blocking the IL-8-CXCR1 axis decreases EGFR phosphorylation, this was more pronounced with the combination of the two drugs (Figure 5).

### Effect of anti-IL8 and anti-CXCR1 neutralization on tumorspheres

**Figure 6:**
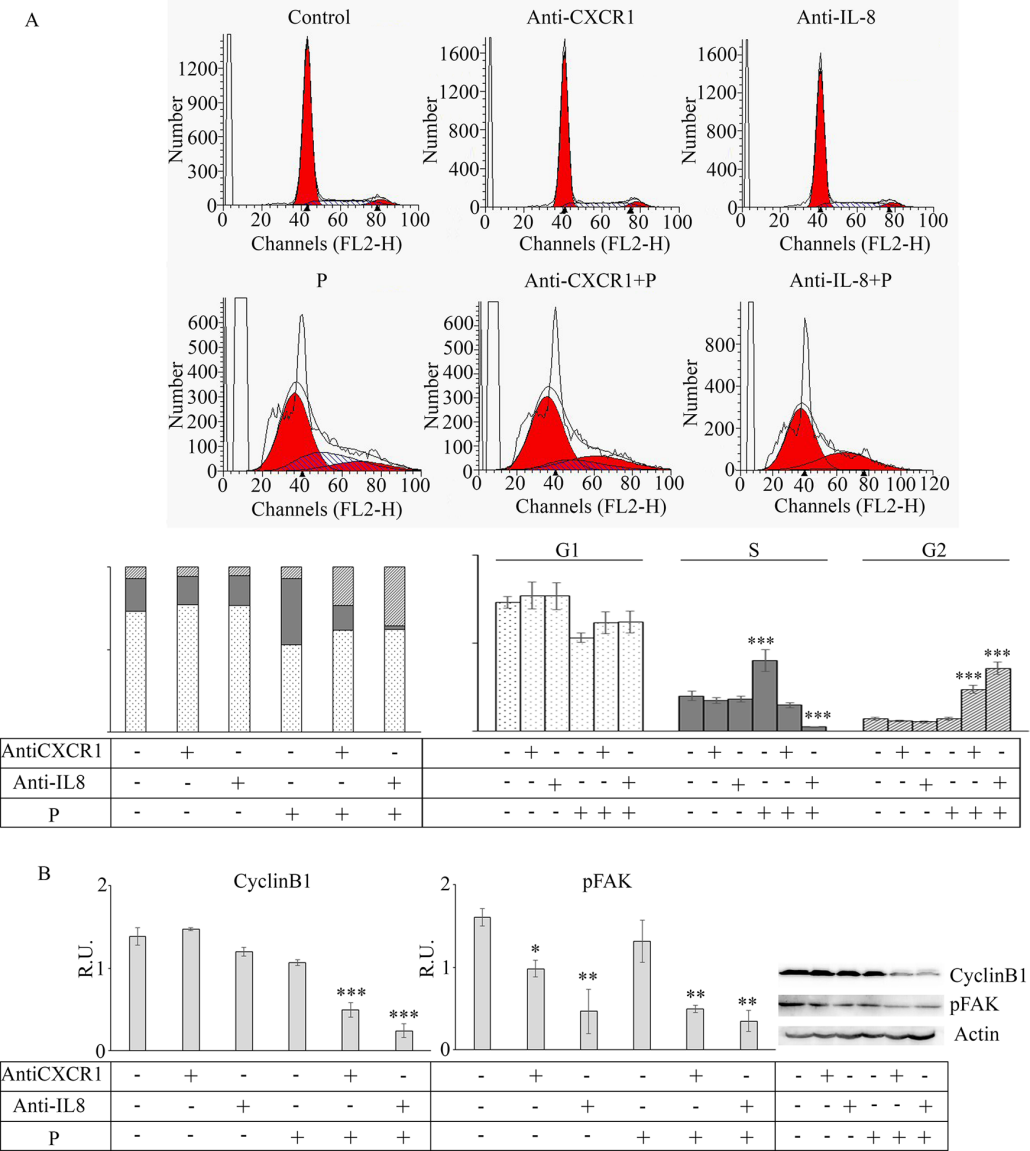
Effect of anti-CXCR1 and anti-IL8 neutralization on mammospheres In **(A)** cytofluorimetric analysis of cell cycle in the presence of the specific antibody administered alone or in combination with paclitaxel (P). Data are mean ± SE of three different experiments. ***p ≤ 0.0005. In **(B)** representative western blotting and relative densitometric analysis, expressed as relative units, for cyclin B1 and p-FAK in the same experimental conditions. Data are mean ± SE of three different experiments. *p ≤ 0.05; **p ≤ 0.005; ***p ≤ 0.0005.

With the aim to confirm that the effects induced by reparixin treatment were mediated by CXCR1, the effect of specific anti-CXCR1 and anti-IL8 antibodies on the cell cycle, cyclin B1 and p-FAK levels were evaluated (Figure 6). Treatment with the two antibodies induced the same effects reported for reparixin treatment on cell cycle (Figure 6A), cyclin B1 and p-FAK (Figure 6B), thus confirming the downregulation of IL-8 -CXCR1 signaling by reparixin.

### *In vivo* experiments

### Effect of reparixin and paclitaxel on metastasis

On the basis of the obtained results, the potential effects of reparixin as single agent or in association with paclitaxel were characterized *in vivo*. Two different experimental sessions that differed for the day of metastasis detection (day 14th, T14, and 21st, T21) have been set up, with the aim to study the temporal progression of metastasis in the brain and to identify a predictive experimental design for testing the potential effects of the drugs.

**Figure 7:**
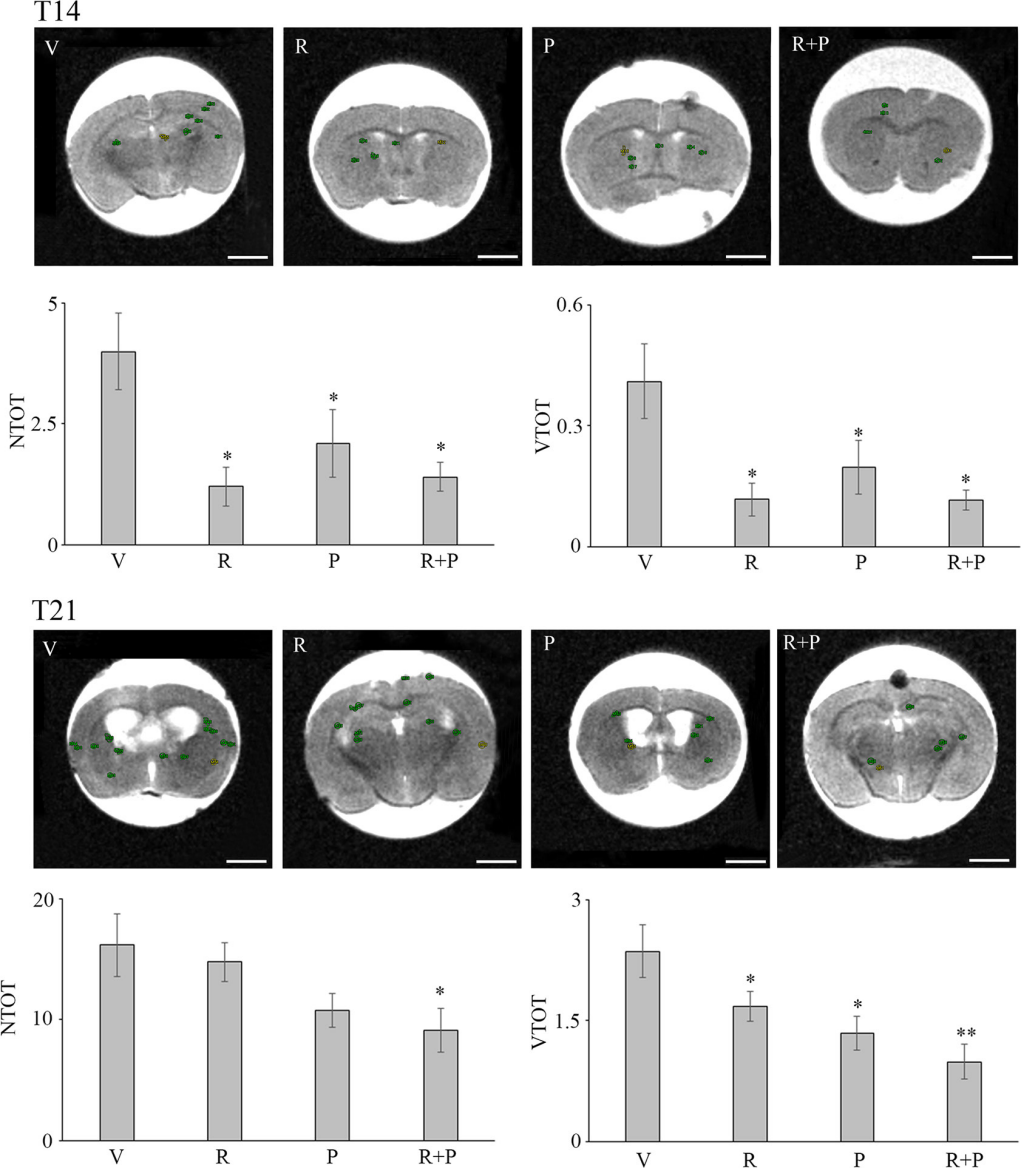
Metastasis formation in nude mice injected with MDA-MB231 cells in the two different experimental models used: the early metastatic model (T14) and a claimed brain tumor occurrence model (T21), both examined by MRI and measured by ROI segmentation Each group consisted of at least 11 animals. Data are mean ± SE. *p ≤ 0.05; **p ≤ 0.01. For each group the top row of each histogram reports representative MRI images, showing ROIs utilized for quantification in control and treated conditions are Bar = 2 mm. VTOT is expressed in μl.

**Figure 8:**
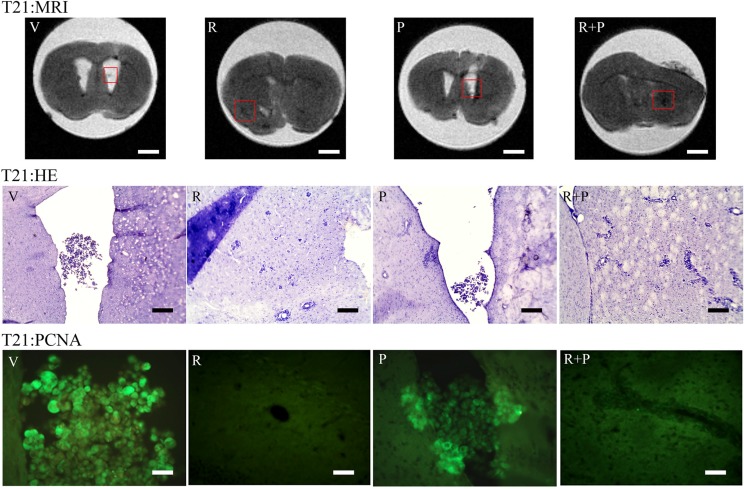
Representative MRI pictures of T21 vehicle and treated animals and HE staining and immunolocalization of PCNA in the same areas identified in the MRI images (red inset) MRI: Bar = 1,7 mm; HE: Bar = 400 μm; PCNA: Bar = 50 μm.

In Figure 7, the total number of masses (NTOT) and total volume (VTOT) parameters for the vehicle (V) (*n* = 19), reparixin (R) (*n* = 13), paclitaxel (P) (*n* = 14) and reparixin + paclitaxel (R + P) (*n* = 14) groups at T14 is reported. It is possible to observe that: (i) upon treatment with reparixin alone, paclitaxel alone or reparixin + paclitaxel administered together, a significant decrease of the number of metastasis is observed; (ii) reparixin alone, paclitaxel alone or reparixin + paclitaxel showed a significant activity in reducing the total volume of metastasis. Figure 7 shows also the NTOT and VTOT parameters for the V (*n* = 11), R (*n* = 12), P (*n* = 12) and R + P (*n* = 12) groups at T21. In this experimental session the drugs treatment started at T7 and lasted until T21. The results obtained showed: (i) a significant inhibitory effect on metastasis number only for the association of reparixin with paclitaxel (group R + P 9 vs 16 for group V); (ii) a statistically significant ability to inhibit the metastasis volume by all the treatment options with a trend of increased inhibitory effect for the combination treatment vs the single treatments (1.67 for R, 1.35 for P, 0.99 for R + P vs 2.36 for V). In the same figure representative MRI images showing the ROI considered for the analysis are also reported. In Figure 8, examples of MRI images in vehicle and animals at T21 are shown. Since the number of masses as well as the dimensions are smaller at T14 than in T21 animals, it was really difficult to envisage the tumor masses by HE or PCNA staining (not shown), probably due to the different thickness of the histology slices with respect to the MRI sections (500 μm versus 20 μm for the histological slices). Fot T21 animals with R or R + P treatments the immunopositivity for PCNA is present at lesser extent than in vehicle or P alone, thus suggesting a decrease of cell proliferation in R-treated animals.

## DISCUSSION

Most of deaths related to breast cancer occur because of metastasis to secondary sites. The cancer stem cell hypothesis considers that only CSCs will initiate and sustain tumor growth. In such model, CSCs are responsible for the metastatic dissemination of tumors. Different lines of evidence indicate that breast cancer stem cells (BCSCs) play an important role in metastasis [18]. BCSCs display increased cell motility, invasion, and overexpress genes that promote metastasis [19–21]. Intracardiac injection of ALDHA1^+^ breast cancer cells, but not unseparated or ALDHA1^−^ cells, into immunodeficient mice, generates multiple metastases. Although a large number of chemotherapeutic agents have been developed which are capable of producing regression of metastatic breast cancers, these tumors usually recur following chemotherapy treatment. Previous studies have suggested that BCSCs are regulated by intrinsic cellular pathways as well as extrinsic signals generated by the tumor microenvironment. Studies by Singh and colleagues on patients [9] elucidated these pathways demonstrating the interactions between the IL-8/CXCR1/2 and HER signaling in the regulation of BCSCs. Moreover, it has previously identified CXCR1, by gene expression profiling, as being overexpressed in cancer cells expressing the stem cell marker ALDH in a series of breast cancer cell lines [11]. CXCR1 is a receptor for the cytokine IL-8, and it has been showed that recombinant IL-8 increased BCSC self-renewal as determined by the ability of these cells to form tumor spheres as well as by increased ALDH expression. Singh et al [9] showed a close association between the IL-8 levels in metastatic fluid and the ability of cells from these samples to generate primary and secondary tumorspheres.

The potential clinical relevance of strategies interfering with the IL-8/CXCR1 axis in BCSC is highlighted by the findings that chemotherapy with taxanes leads to the release of IL-8 and to the increase in the BCSC population [11, 22–23] both *in vitro* and *in vivo*, whereas the administration of anti-CXCR1 agents or anti-IL-8 blocking mAb reduces BCSC percentage in tumors [11, 22–23]. With the aim to improve the knowledge on IL-8 inhibitors and on their possible use as anti-cancer drugs for breast metastasis, in this study the effects of reparixin, a clinical grade CXCR1/2 inhibitor, administered alone or in combination with paclitaxel, were assayed *in vitro* on purified mammary tumorspheres from MDA-MB231 cell line and *in vivo* in a model of breast metastasis to brain, utilizing two different experimental conditions.

The cellular model used in this study was the triple negative MDA-MB231 cells, that were not extensively studied in previous experiments utilizing reparixin. This cell line is highly aggressive and does not undergo to apoptosis via Fas-ligand upon paclitaxel challenge (data not shown), as described for other breast cancer cell lines [11]. The majority of studies on breast cancer metastasis used MDA-MB231 cells. This cell line represents the claudin-low/mesenchymal subtype, which overexpresses stem cell-enriched genes [24] and has a natural tendency to metastasize to brain and lungs. In addition, this is the breast cancer cell line with the highest ALDH1 expression of [25]. Moreover, in this work the effects of reparixin were investigated in highly purified mammospheres derived from the cell line. The *in vitro* model, purified mammospheres (enriched in CXCR1 as well as of ALDHA1), allowed to demonstrate that reparixin, administered at different concentrations, determined a dose-dependent effect on cell viability, more apparent when associated with paclitaxel. Reparixin alone was able to decrease the active form of the focal adhesion protein (p-FAK), in keeping with previous findings [11], and β-catenin, while paclitaxel alone appears to promote apoptosis. Focal adhesion kinase (FAK) is a cytoplasmic tyrosine kinase identified as a key mediator of intracellular signaling by integrins in the regulation of different cellular functions in a variety of cells [26]. Early studies showed that stimulation of FAK activation and phosphorylation by oncogenic transformation provided a plausible molecular mechanism for anchorage-independent growth of cancer cells, one of their major hallmark [27]. Numerous studies have then linked FAK-mediated signaling pathways to breast and other cancers as well as a variety of different biological and disease processes. Upon its activation by integrin-mediated cell adhesion or other stimuli, FAK becomes associated with several molecules containing SH2 domain including Src and p85 subunit of PI3K [28–29] through its autophosporylated Y397 residue. FAK association and activation of PI3K through autophosphorylated Y397 leads to increased production of 3′-phosphorylated phospholipid [30], which can activate Akt kinase inhibiting apoptosis by regulating various cell death machinery proteins [31–32]. These observations appear relevant in view of the decreased PI3K, p-Akt and p-FAK levels promoted by reparixin in our experimental conditions. Very recent studies by several groups showed that ablation of FAK suppressed mammary tumorigenesis and progression in mouse models of breast cancer [26, 33–36]. Furthermore, these studies demonstrated that deletion of FAK reduced the pool of BCSCs in primary tumors developed in FAK conditional knockout mice [34]. These studies suggest a causal role of FAK in promoting breast cancer *in vivo* and also give further support for the CSC hypothesis. Finally, it has been reported that the inhibition of FAK *in vitro* and *in vivo* decreased self-renewal capacity, by decreasing the levels of Wnt3a and β-Catenin, thus demonstrating a novel FAK-Wnt axis regulating BCSC activity and indicating the FAK-Wnt axis as promising target to eradicate self-renewal capacity and progression of human breast cancers [15]. In agreement, in our experiments reparixin alone was able to decrease mammosphere size, p-FAK and β-catenin levels, thus suggesting that it may also interfere with the FAK-Wnt axis.

Moreover, it appears that some parameters were modulated by the two drugs, administered together, in a synergistic manner, particularly the cell cycle arrest in G2/M phase and the decrease of cyclin B1 levels as well as the induction of the senescence pathway. Cellular senescence is the process that leads to terminal growth arrest induced by unrepairable double strand DNA damage. Activation of the oncogenes as well as inhibition of the tumor suppressor genes were shown to contribute to senescence induction and senescence is considered as a natural antitumor barrier that acts at the early stages of cancerogenesis to stop the proliferation of transformed cells. However, tumor cells still remain sensitive to induction of senescence, for example during chemio- or radiotherapy. Thus, induction of cancer cell senescence, similarly to apoptosis, is considered to restrain tumor growth and thus contribute to effectiveness of anticancer therapy. The senescent cells, although do not proliferate, remain viable and metabolically active [37]. Cellular senescence is now increasingly recognized as a possible outcome for the treatment of human tumours because it is executed by cells in response to therapeutic treatments, such as drugs and irradiation; therefore, it has recently emerged that senescence could be a potential alternative outcome for tumour therapy *in vivo* [38].

An additive effect was observed on other parameters such as mammosphere numbers and cell viability. The involvement of IL-8 and the specific effects of reparixin as the single agent or in combined treatment were supported by the use of the specific antibodies against IL-8 or CXCR1.

The specific involvement of reparixin in the counteraction of the migrative potential of stem cells, by decreasing p-FAK observed in our experimental *in vitro* conditions, was further supported by the data analysis of the *in vivo* experiments.

We show here that *ex vivo* MRI at 2.35 T allows the characterization of breast cancer cells brain metastasis in a mouse model. In agreement with other Authors [39], we found that there is an increase in size and number of metastatic masses over time. On this basis, we choose two different experimental conditions. One, an advanced metastatic model, where tumor was allowed to grow for 7 days and then the treatments started; the other, an early model of metastatic cell migration, where the tumor cells were injected and the treatments started simultaneously.

Upon 14 day of treatments, different considerations may be done on the different experimental conditions. In fact, in animals starting treatments after 7 days from the tumor cell injection, the reparixin or paclitaxel treatment and the combined treatment show a significant activity in reducing the total volume of metastasis, while only the combined treatment shows a significant activity on the total mass number. This may be due to the fact that, over 7 days, cells reached and invaded the brain parenchyma and the only possible activity of the single reparixin or paclitaxel treatment was the decrease tumor cell proliferation, by, as indicated *in vitro*, decreasing Δ-catenin and p-FAK, CFSE, for reparixin, or by inducing apoptosis for paclitaxel and to their incapability at this time-point to decrease tumor invasion. The combined treatment was able to induce both phenomena, decreasing cell proliferation and inducing apoptosis, thus resulting in a decrease of the total mass number. This hypothesis is further supported by PCNA staining where it is possible to observe that reparixin alone and the combined treatment induce a decrease of PCNA-positive nuclei, thus indicating a decrease of proliferation.

This seems to be in agreement with the findings obtained in the other experimental condition, i.e. animal injected and simultaneously treated with reparixin or paclitaxel or reparixin + paclitaxel. In fact, in this case the treatment with reparixin alone, paclitaxel alone or reparixin + paclitaxel shows a significant activity in reducing the number of metastasis, probably due to an immediate effect on cell migration by reparixin and cell death by paclitaxel. All treatments determine a significant reduction of the total number and volume of metastasis, in agreement with the hypothesis of fewer cells reaching brain parenchyma and with the described effect of the single drug. This result may indicate that reparixin is able to decrease the number of metastasis by counteracting at this time point BCSC migration and proliferation by decreasing p-FAK and Δ-catenin. On the other hand, the effects of paclitaxel are mainly due to apoptosis promotion. The combination shows the additive effects of the single drugs. It is worthnoting that the results obtained in the combinatorial group are not as robust as observed in the *vitro* experiments. We interpret this apparent discrepancy between *in vitro* and *in vivo* results as a consequence of the intrinsic variability of the metastases model. In fact, two important factors affecting the *in vivo* evaluation of the drug combination in the brain metastasis model, i.e. drugs distribution and tumor volume at the start of the treatment, should be considered. In fact, it is generally known that anticancer drugs are poorly permeant to blood brain barrier (BBB) and permeability strictly depends on the level of damage introduced during brain metastasis derivation. That means that as much BBB has been broken during breast cancer cells invasion as much drug has been distributed. These factors may have a different impact on paclitaxel and reparixin distribution and can account for the higher variability in results observed *in vivo*.

Considering the significant morbidity and mortality caused by brain metastases, several therapeutic approaches have been pursued [40]. From this standpoint, CSC represent a potential target as they are responsible for disease relapse and metastasis [9]. Administration of a CSC-targeting agent in patients at high risk for developing brain metastases (i.e., HER2 + and TNBC patients) could readily test this hypothesis. However, the adjuvant setting would pose strong challenges in terms of patient number and time of observation required [41–42]. Selected patient groups are at even higher risk of developing BCBM (breast cancer brain metastasis) in a shorter timeframe and would allow a faster evaluation of the potential of a new drug on the reduction of BCBM incidence [42]. Such hypothesis is being tested in a clinical trial of reparixin plus paclitaxel in women with metastatic TNBC (with no documented brain metastases) relapsed after (neo) adjuvant treatment (NCT02370238). While in the preventive setting there is no requirement for penetration of an intact BBB by the drug, which is expected to inhibit the metastatic potential of csc outside of the central nervous system, the medical treatment of established BCBM in combination with other agents would require crossing of an allegedly broken BBB. This would also heavily rely on the antitumor activity of the partner molecule (e.g., chemotherapy) in order to rapidly shrink BCBM and reduce patient's symptoms, therefore the combination requires careful selection of an active drug based upon the tumor type being treated. The data presented here suggest that such combination strategy can be translated into future clinical trials in dedicated patient populations.

## MATERIALS AND METHODS

### Cell line and reagents

Human breast cancer cell line MDA-MB231 was obtained from the European Collection of Cell Cultures (ECACC). Dulbecco's modified Eagle's (DMEM), Dulbecco's modified Eagle's medium/F-12 medium (DMEM-F12), Fetal Bovine Serum (FBS), penicillin/streptomycin, glutamine, formaldehyde, paraformaldehyde, triton X-100, Phosphate Buffered Saline (PBS), Bovine Serum Albumin (BSA), poly-L-lysine, dapi, trypan blue, paclitaxel, Dimethyl Sulfoxide (DMSO), ethanol, propidium iodide, Nonidet-P40, RNAse, sodium deoxycholate, Sodium Dodecyl Sulphate (SDS), Tween 20, Igepal CA 630, Ethylenediamine tetraacetic acid (EDTA), phosphatase inhibitor cocktail 2, protease inhibitor cocktail, ethylenediamine tetraacetic acid (EDTA), acrylamide/bis-acrylamide, *Tris* (hydroxymethyl)aminomethane (Tris), hydrogen chloride (HCl), sodium chloride (NaCl), xylazine hydrochloride, Hank's Balanced Salt Solution (HBSS), and sucrose were all purchased from Sigma Chemical CO (St. Louis, Mo, USA). B27 supplement were from Life Technologies (Paisley, Scotland, UK), Epidermal Growth Factor (EGF) and Fibroblast Growth Factor (FGF2) were from Peprotech (Rocky Hill, NY, USA). reparixin (DF1681Y) was provided by Dompé Farmaceutici Spa (L'Aquila, Italy).

Primary antibodies anti-ALDH1A1, anti-p-FAK, anti-p-Akt, anti-ABCG2 and anti-PCNA were purchased from Sigma Chemical CO, anti-CXCR1 (clone 42705), anti-CXCR2 (clone 48311) and anti-IL-8 (clone 6217) were from R&D Systems Inc. (Minneapolis, USA). Antibodies anti-Cyclin D1, anti-PI3 Kinase, p27 and anti-P-FOXO3A were purchased from Abcam (Cambridge, MA, USA), anti-Cyclin B1 was from Acris Antibodies (San Diego, CA, USA), anti-NFkB and anti-P-EGFR were from Santa Cruz Biotechnology, Inc. (Santa Cruz, CA, USA), and anti-β catenin was from Zymed (San Francisco, CA, USA). Phalloidin-tetramethylrhodamine isothiocynate (Phalloidin TRITC) was purchased from Sigma Chemical CO and human monoclonal CD44-Phycoerythrin antibody was from Miltenyi Biotec Inc. (Auburn, CA, USA). AlexaFluor 488 anti-mouse or anti-rabbit IgG secondary antibodies were purchased from Molecular Probes (Life Technology, Carlsbad, CA, USA) and peroxidase conjugated anti-mouse or anti-rabbit IgG secondary antibodies were from KPL (Gaithersburg, USA). Aldehyde dehydrogenase Based Cell Detection Kit was from StemCell Technologies (Durham, NC), CellTiter 96^®^ AQ_ueous_ non-radioactive cell proliferation assay from Promega Corporation (Madison, USA), bicinchoninic acid (BCA) protein assay kit from Pierce (Rockford, IL, USA) and CellTrace^™^ CFSE cell proliferation kit was purchased from Molecular Probes (Life Technology, Carlsbad, CA, USA). Vectashield mounting medium was required to Vector Laboratories (Burlingame, CA, USA), non-fat dry milk was from Bio-Rad Laboratories (Hercules, CA, USA), SuperSignal West Pico Chemiluminescent Substrate and Nunc low adherent culture flasks were purchased from Thermo Scientific (Rockford, IL, USA). Immobilon-P Transfer Membrane (PVDF) was required to Millipore Corporation (Billerica, MA, USA), O.C.T. was from Sakura (St. Torrance, CA, USA), hematoxylin-eosin was from Bio-Optica SpA (Milano, Italy) and gelatin glycerin was from Electron Microscopy Sciences (PA, USA). Ketamine was purchased from Veter-Zoo S.r.l. (Perugia, Italy). NUNC low adherent flasks were from Thermo Fisher Scientific Inc. (Walthman, MA, USA). All chemicals were of the highest analytical grade.

### Cell culture

MDA-MB231 were plated at 30.000 cells/cm^2^ in DMEM supplemented with 10% FBS, 0.1 mg/ml penicillin/streptomycin, 2 mM glutamine, at 37^°^C, in a humidified 95% air-5% CO_2_ atmosphere.

### Mammospheres isolation and purification

MDA-MB231 cells were plated at 1000 cells/ml in DMEM-F12, containing 100 units/ml penicillin/streptomycin, 2 mM glutamine, 2% B27 supplement and 20 ng/mL EGF and 40 ng/mL FGF2. Cells were cultured in NUNC low adherent culture flasks, at 37^°^C, in humidified 95% air-5% CO_2_ atmosphere. Primary tumourspheres were dissociated mechanically and cultured for several passages (clonal selection).

### Flow cytometer analysis

To evaluate the expression panel of several stemness markers such as CXCR1, CXCR2, ABCG2, ALDH1A1, MDA-MB231 cells and mammospheres were dissociated and the single-cell suspensions (1 × 10^6^ cell/tube) were kept, for 15 minutes at Room Temperature (RT), with 2% formaldehyde in PBS. For detection of ALDH1A1 the cells were permeabilized with 0.1% Triton-X-100, for 5 minutes at RT. Cells were washed with PBS and then incubated, for 1 hour at RT, with selected primary antibodies: monoclonal anti-CXCR1 (1:100), anti-CXCR2 (1:100), anti-ABCG2 (1:50) and polyclonal anti-ALDH1A1 (1:200), all diluted in PBS containing 4% BSA. After washing with PBS, the cells were incubated for 1 hour at RT, with secondary AlexaFluor 488-conjugated anti-mouse or anti-rabbit IgG antibodies diluted 1:2000 in PBS containing 4% BSA. The expression of CD44 antigen was evaluated in purified mammospheres, incubating cells with monoclonal anti-human Phycoerythrin-conjugated CD44 antibody. Cells were then washed with PBS and the population of interest was gated according to its Forward Scatter (FSC)/Side Scatter (SSC) criteria. 10.000 events were acquired for each sample and analyzed by CellQuest software. (Becton Dickinson Biosciences, BD, San Diego, CA).

### Aldefluor assay

ALDH enzymatic activity in control MDA-MB231 cell line and in control mammospheres was detected using Aldehyde Dehydrogenase Based Cell Detection Kit (StemCell Technologies, Duham, NC), according to manifacture's protocol. Flow cytometry data was acquired and analyzed by CellQuest software (BD Biosciences) using a FACS Calibur flow cytometer.

### Cell viability

For cell viability evaluation in mammospheres, single-cells were plated at 10000 cells/cm^2^ and after 24 hours treated with different concentrations of reparixin (1 μM, 10 μM and 50 μM) alone and in the association with 5 nM paclitaxel. The effect of drugs on cell viability was estimated after 72 hours of treatment by counting cells with trypan blue.

### Pharmacological treatments

Reparixin (Bacth No. 117410001S) was prepared in PBS (pH 7.9–8.1) at a concentration of 15 mg/ml, paclitaxel was dissolved in 100% DMSO at a concentration of 50 mg/ml. For all the parameters analysed, single-cells from mammospheres were plated at a density of 10000 cells/cm^2^ and after 24 hours were treated with: reparixin (10 μM), paclitaxel (5 nM), their association, the anti-CXCR1 and anti-IL8 antibodies (20 μg/ml) alone and in association with paclitaxel (5 nM), for 72 hours.

### Cell cycle and apoptosis analysis by FACS

For cell cycle and apoptosis analysis, mammospheres untreated and treated with reparixin, anti-CXCR1 and anti-IL8 antibodies alone and in association with paclitaxel for 72 h were collected, dissociated, washed twice with icecold PBS and fixed in 70% ethanol at 4°C for 30 min. Then, fixed cells (1 × 10^6^ cells/ml), were washed twice with ice-cold PBS and stained with solution containing 50 μg/mL propidium iodide, 0.1% Nonidet-P40 and RNase A (6 μg/1 × 10^6^ cell) for 30 min in the dark at 4^°^C. Cell cycle phase-distribution was analysed by a flow cytometry system. Data from 10000 events per sample were collected and analysed using FACS Calibur (Becton Dickinson) instrument equipped with cell cycle analysis software (Modfit LT for Mac V3.0). Apoptotic cells were determined by their hypochromic subdiploid staining profiles and analysed using Cell Quest software (BD Biosciences, San Diego, CA).

### Protein assay

Protein were assayed by the Pierce BCA Protein Assay kit (Pierce, Rockford, IL, USA) reading the absorbance at 562 nm.

### Western blotting

Control and treated mammospheres were lysated in ice-cold RIPA buffer (0.5% sodium deoxycholate, 0.1% SDS, 1% Igepal CA630, 5 mM EDTA in PBS, 10 μl/ml both Protease and Phosphatase Inhibitor Cocktails) sheared through a 22-gauge needle and centrifuged at full-speed (Eppendorff microfuge 5418), at 4^°^C, for 30 minutes. Protein lysates (30–50 μg) were separated on 12–15% SDS-polyacrilamide gel and electroblotted onto PVDF. Nonspecific binding sites were blocked by 5% non-fat dry milk in Tris buffered saline (TBS: 20 mM Tris-HCl, pH 7.4, containing 150 mM NaCl) with 0.1% Tween 20 (TBS-T) for 30 minutes at RT. Membranes were then incubated overnight at 4°C with the following primary antibodies, all diluted with TBS-T: rabbit anti-PI3 Kinase (1:500), p27 (1:5000), anti-Cyclin B1 (1:200), anti-P-FAK (1:500), anti-P-Akt (1:500), anti-P-EGFR (1:200) and mouse anti-β catenin (1:500) and anti-NFkB (1:200). All antibodies were diluted with TBS containing 0.1% Tween-20 (TBS-T) and 5% non fat dry milk. As secondary antibodies, peroxidase conjugated anti-rabbit or anti-mouse IgG (1:10000), in TBS-T containing 5% non fat dry milk, were used for 1 hour, at RT. Immunoreactive bands were visualized by enhanced chemiluminescence (ECL, Biorad, Hercules, CA, USA), according to the manufacturer's instructions. The relative densities of the immunoreactive bands were determined and normalized with respect to actin, using a semiquantitative densitometric analysis with the ImageJ Software [43]. Values were given as relative units (RU).

### Carboxyfluorescein diacetate succinimidyl ester (CFSE) and cell proliferation

Cell proliferation is evaluated by the CellTrace^™^ CFSE cell proliferation kit according to manufacturer protocol. Briefly, mammospheres were dissociated and incubated with PBS containing 5 μM CFSE/1 × 10^6^ cells and 0.1% BSA, for 15 minutes, at 37^°^C. The reaction was stopped with cold culture medium and after washing with medium, cells were seeded at 10000 cells/cm^2^ and treated, after 24 hours, with 10 μM reparixin alone and in association with 5 nM paclitaxel.

After 72 hours, mammospheres were allowed to adhere on poly-L-lysine coated glass coverslips and fixed in 4% paraformaldehyde in PBS, for 10 minutes, at RT. Cell nuclei were stained with DAPI (0.5 μg/ml). Coverslips were mounted with vectashield mounting medium and examined at a Leica TCS SP5 confocal microscope (Mannheim, Germany).

### Immunofluorescence

To evaluate the expression panel of CXCR1, CXCR2, ABCG2, ALDH1A1, mammospheres were allowed to adhere on poly-L-lysine coated coverslips (15 μg/ml) and fixed in 4% paraformaldehyde in PBS, for 10 minutes at RT. Non-specific binding sites were blocked with 4% BSA in PBS (blocking solution), for 10 minutes at RT. For detection of ALDH1A1, mammospheres were permeabilized with 0.1% Triton-X-100, for 5 minutes, at RT. Cells were washed with PBS and then incubated, overnight at 4^°^C, with the following antibodies: mouse anti-CXCR1 (1:100), anti-CXCR2 (1:100), anti-ABCG2 (1:50) and rabbit anti-ALDH1A1 (1:200), all diluited in PBS containing 4% BSA. For actin microfilaments detections, control and treated mammospheres were permeabilized with 0.1% Triton-x-100, for 5 min, at RT and incubated, for 30 minutes, at RT, with Phalloidin TRITC diluted 1:5000 in blocking solution, and for the analysis of the immunoreactivity to cyclin B1 and FOXO3A, control and treated mammospheres were incubated with rabbit anti-cyclin B1 (1:200) and anti-P-FOXO3A (1:1000) antibodies, overnight, at 4^°^C. After washing with PBS cells were incubated, for 30 minutes at RT, with AlexaFluor 488 anti-mouse or anti-rabbit IgG secondary antibody, diluted 1:2000 in blocking solution. Controls were performed by omitting the primary antibody. Cell nuclei were stained with DAPI (0.5 μg/ml). Coverslips were mounted with Vectashield Mountin Medium and examined at a Leica TCS SP5 confocal microscope (Mannheim, Germany).

### Mammospheres and single cells number

Control and treated mammospheres were harvested and the numbers of mammospheres and single cells was counted in 96 well microplate by trypan blue exclusion.

### Dimension analyses of mammospheres

Upon pharmacological treatment mammospheres were collected and allowed to adhere on poly-L-lysine coated coverslip and fixed in 4% paraformaldehyde in PBS, for 10 minutes at RT. Cell nuclei were stained with DAPI (0.5 μg/ml). Coverslips were mounted with Vectashield Mounting Medium and examined and counted at a Leica TCS SP5 confocal microscope.

### *In vivo* experiments

### Animals and ethical approval

Nude Balb/c mice (female, 4 weeks od) (Charles River Laboratories, Calco, Italy) were used for the study. The animals were caged (4/cage) under clean conditions at the animal facilities of the University of L'Aquila (Italy). Mice had free access to pellets and water during a 12:12-hour dark-light cycle. All the environmental conditions, as well as all the procedures adopted throughout the study for housing and handling the animals were conducted in strict compliance with national and international laws and policies (EEC Council Directive 86/609, OJ L 358, 1, Dec. 12,1987; Italian Legislative Decree (Gu n. 61,14/03/2014); NIH guide for the Care and Use of Laboratory Animals, NIH Publication No. 85–23, 1985), and were approved by the Institutional Review Board of the University of L'Aquila.

Each animal was randomly distributed into experimental groups. All animals were sacrificed by perfusion.

Nude BALB/c mice were anesthetized intraperitoneally with 100 mg/Kg ketamine 10 mg/Kg xylazine hydrochloride and injected in the internal carotid artery with 250.000 MDA-MB-231 cells in 100 μl of HBSS. Before injection, cell viability of MDA-MB-231 was evaluated by MTS assay.

### Time course of tumour formation

A time course experiment was performed in order to follow the metastasis development. Mice injected with tumour cells, were sacrificed at 14 (T14) and 21 days (T21) after the injection, respectively.

### Evaluation of the effects of drug treatments

To evaluate the effect of reparixin and paclitaxel on brain metastasis (development), two different experiments were set up. In the first set, mice, were treated with drugs the same day of tumour cells injection and sacrificed after 14 days (T14 groups). Reparixin was dissolved in Phosphate Buffer Solution (PBS) pH 7.9–8.1 at the concentration of 15 mg/ml and administered at 45 mg/Kg (100 μl). Paclitaxel was prepared in 100% DMSO (50 mg/ml) and administered, i.p, at the dose of 10 mg/Kg. Mice were treated with: vehicle (V-T14: PBS pH = 7.9–8.1 s.c. twice daily, every 8 hours, and DMSO i.p. once a week); reparixin (R-T14: 45 mg/kg s.c. twice daily, every 8 hours); paclitaxel (P-T14: 10 mg/ml i.p. once a week); reparixin + paclitaxel (R + P-T14: 45 mg/kg s.c. twice daily, every 8 hours and 10 mg/ml i.p. once a week). In the second set of experiments, mice, were treated 7 days after injection of tumour cells (T21 groups), with: vehicle (V-T21: PBS pH = 7.9-8.1 s.c. twice daily, every 8 hours, and DMSO i.p. once a week); reparixin (R-T21: 45mg/kg s.c. twice daily, every 8 hours); paclitaxel (P-T21: 10 mg/ml i.p. once a week); reparixin + paclitaxel (R + P-T21: 45 mg/kg s.c. twice daily, every 8 hours and 10 mg/ml i.p. once a week). In both experimental set up each group consisted at least of 11 mice and the treatment lasted 14 days.

At the end of the treatment protocols, the mice were anesthetized with ketamine/xylazine hydrochloride i.p. and intracardiacally perfused with 10% formalin solution neutral buffered. Brains were dissected out and transferred in formalin for MRI imaging.

### MRI

The samples for the MRI analysis consisted of *ex vivo* adult Balb/C nude mice brains (weight between 300 and 490 mg) immersed in 15 ml of 4% formaldehyde in 0.1 M PB at pH 7.4. In this study a 2.35 T Bruker Biospec scanner comprising a superconducting magnet BC24/40 with a free bore of 400 mm and static field homogeneity in a 60 mm diameter spherical volume (DSV) of 0.05 ppm (half height) was used. A gradient system BGA26 with a free bore of 257 mm provided a gradient strength of 21 kHz/cm at maximum current of 100 A (corresponding to a maximum of 50 mT/m). The minimum inductive rise time of the gradients was about 160 microseconds. The linearity of the gradients was better then 3% in a 60 mm DSV. A shim set BS40 provided up to eight spherical harmonic terms with a maximum error less then 3%. The scanner was equipped with a transmit/receive birdcage radio frequency volume coil (8 rungs, internal diameter 62 mm, external diameter 120 mm, length 111 mm, Doty Scientific Inc. USA). The RF coil was tuned at the proton frequency of 100.4 MHz. The radio frequency amplifier provided a peak power of 1 kW capable of generating the 90^°^ and 180^°^ RF pulses. The scanner was interfaced with an HP XW4600 workstation running a Linux operating system (RH WS4). The workstation comprises the Bruker Paravison 4.0 software for data acquisition and processing.

### MRI data acquisition

At a variable time from the sacrifice, after storage at 4^°^C, for each measurement session four brains were positioned in a plastic holder at the centre of the magnet for MR image acquisition. Once in place and loaded with the plastic holder containing four samples the RF coil was tuned and matched using first a network analyzer (Agilent E5061A) and then the “wobble” procedure from Bruker. For all measurements the tuning frequency was within 0.1 MHz from the centre and the matching better then −15 dB. First, a single pulse sequence (NSPEC) was applied to observe the NMR signal (TR = 4500 ms; flip angle = 30^°^; tp = 100 μs; BW = 3kHz; 2048 points; spectral width 30 ppm; resolution 0.73 Hz/point; NEX = 1; TACQ = 700 ms). This sequence was used to calibrate the: RF pulse, receiver gain, transmit flip angle and auto-shim settings. Second, to check the position and orientation of the samples fast scout gradient echo (GE) images (FLASH) were acquired with the following parameters: TR = 700 ms; TE = 30 ms; FOV = 66 mm × 66 mm; 128*128 pixels; 516 μm in plane resolution; slice thickness = 1.6 mm; slices number = 15 (13 axial, 1 sagittal and 1 coronal); NEX = 1; TACQ = 11 min. Then, fast two-dimensional (2D) axial and coronal GE T2-weighted (T2-W) images (GEFC protocol), covering the whole brain, were acquired with the following conditions: TR = 2700 ms; TE = 47 ms; FOV = 27 mm × 27 mm; 128*128 pixels; 210 μm in plane resolution; slice thickness = 500 μm; inter-slice distance 1 mm; slice number = 23; NEX = 1; TACQ = 6 min. These images (axial and coronal planes) were used to verify the absence of air bubbles attached to the brains or the sample tube that could create significant image artefacts. In such cases the sample tube was taken out and the preparation procedure was repeated again.

Finally, to assess anatomical details and characterize the presence of masses in the brain, 2D high-resolution (HR) axial GE T2-W images, covering the whole brain, were acquired with the following parameters: TR = 4700 ms; TE = 50 ms; flip angle = 75^°^;FOV = 25 mm × 25 mm; 256*256 pixels; 98 μm in plane resolution; slice thickness = 500 μm; slices number = 35; NEX = 16; TACQ = 5 hours and 20 min. These images were used for metastasis assessment, as explained in the next section.

### Manual ROI segmentation

To extract information about the presence of metastasis, a region-of-interest (ROI) manual segmentation method was used. For each brain the high-resolution T2-W MR images comprising up to 25 axial slices were visualized and analyzed with the software Paravision 4.0. To identify the metastasis each slice was analyzed with the tool “image contrast” by adjusting the grey values within at least three level intervals, such as to isolate the suspected ROIs characterized by low intensity. Then the position, shape and dimension of the ROIs were compared with the normal anatomy of that slice, as reported in the Paxinos Atlas [44].

Thus, from the analysis of the slices the following two parameters were extracted: the total number of metastasis (NTOT) present in the brain and the total volume of the metastasis (VTOT) in μl. It is worth noting that since the MR images are acquired with a two-dimensional modality, this may give an over-estimation of the total number of masses NTOT. The entity of this effect will depend on the relative size of the slice thickness (in our conditions 500 μm) and the size of the metastasis that depends on the time post injection. To evaluate the entity of this over-estimation effect we developed a Matlab algorithm that associates masses in contiguous slices as a single mass when the distance of their centers is smaller than the average of their pseudo-radius. The method was applied to a subset of MR images and it was found that the over-estimation is less than 14%. Given this relatively small value we assumed the measured parameters (NTOT, VTOT) to be robust.

To avoid the contribution of false positive values, due to the intrinsic noise present in the high-resolution T2-W images, a filtering procedure, based on the histogram analysis of a control group (*n* = 5 mice) that did not received the inoculation of the breast cancer cells, was adopted for all the images.

### Histology and immunofluorescence

After MRI, brains were processed for histological analysis. Briefly, brains were transferred in 30% sucrose for 2–3 days, then embedded in O.C.T. and frozen. Serial Coronal cryostat sections (20 μm thick) were processed for histological (hematoxylin-eosin staining) and immunofluorescence analyses. Observations and photography of histological sections were carried out with Stereozoom Leica S8APO microscope equipped with digital camera EC3 (Mannheim, Germany) Image acquisition was performed with Leica Acquire 1.0 program (Mannheim, Germany).

For immunofluorescence analysis, non-specific binding sites were blocked with PBS containing 4% BSA and 0.25% Tween-20, for 2 hours at RT and brain sections were incubated, overnight at 4^°^C, with antibody rabbit anti-PCNA (1:200), diluted in PBS containing 4% BSA. After washing with PBS slides were incubated for 2 hours at RT, with AlexaFluor 488anti-mouse or anti-rabbit IgG secondary antibody, diluted 1:2000 in PBS containing 4% BSA. Controls were performed in parallel by omitting the primary antibody. Coverslips were mounted with Vectashield Mountin Medium and examined with a florescence microscope (Zeiss Axioplan 2, Gottingen, Germany) equipped with Leica DFC 350 FX camera. Image acquisition was performed with Leica IM500 program (Mannheim, Germany).

### Statistical analysis

For the *in vitro* experiments, samples were processed by SPSS software (Statistical Package for Social Sciences, v. 11.0, Tokyo, Japan). Statistical analysis of two population means was performed by the unpaired Student's *t* test, while statistical differences comparing multiple means were analyzed by the analysis of variance (ANOVA) followed by Scheffe's post hoc test analysis. *P < 0.05; **P < 0.005, **P < 0.0005. Data were expressed as Mean ± SE of 3 separate experiments. For the *in vivo* experiments, the measured parameters (NTOT, VTOT) were imported in STATISTICA (version 8.0, StatSoft Inc. 2007) in order to perform descriptive statistical analysis and ANOVA to analyze the differences between groups. Results were considered statistically significant if *p* < 0.05 (*) or *p* < 0.01 (**). Newmann-Keuls post hoc tests were further utilized. All data were expressed as Mean ± SE.
